# The New TLC Method for Separation and Determination of Multicomponent Mixtures of Plant Extracts

**DOI:** 10.1155/2016/1813581

**Published:** 2016-02-14

**Authors:** Elżbieta Matysik, Anna Woźniak, Roman Paduch, Robert Rejdak, Beata Polak, Helena Donica

**Affiliations:** ^1^Department of Analytical Chemistry, Medical University of Lublin, Chodźki 4A, 20-093 Lublin, Poland; ^2^Department of General Ophthalmology, Medical University of Lublin, Chmielna 1, 20-079 Lublin, Poland; ^3^Institute of Microbiology & Biotechnology, Department of Virology & Immunology, Maria Curie Sklodowska University, Akademicka 19, 20-033 Lublin, Poland; ^4^Department of Physical Chemistry, Medical University of Lublin, Chodźki 4A, 20-093 Lublin, Poland; ^5^Department of Biochemical Diagnostics, Medical University of Lublin, Staszica 16, 20-081 Lublin, Poland

## Abstract

The new mode of two-dimensional gradient thin layer chromatography (MGD-2D TLC) has been presented. Short distance development of sample in the first dimension leads to formation of the preconcentrated narrow zones. They are consecutively separated in the second dimension with the mobile phase gradient in several steps of development until the eluent reaches the further end of the chromatographic plate. The use of the above-mentioned technique allows isolating and then identifying the compounds of various polarity from the multicomponent mixture. The practical application of two-dimensional gradient thin layer chromatography has been performed for isolation of the two plant (*Juniperus* and* Thymus*) oils components as the examples of test mixtures. The experiments have been carried out with the use of silica gel plates as well as a normal phase condition. The results of solute separation with isocratic one-dimensional thin layer chromatography system have been compared with those of two-dimensional gradient system. It has been observed that application of the latter mode leads to almost triplicated number of zones in comparison with the former one. It is purposeful to apply the proposed mode to control the purity of the dominant component or components of the mixture.

## 1. Introduction 

The separation and determination of compounds in multicomponent mixtures, that is, plant extracts, are usually performed with the use of high performance liquid chromatography, HPLC (e.g., [1, 2]), or planar chromatography (e.g., [3–5]). The sample complicity often involves application of two-dimensional system in both techniques.

The comparison of solute and standard retentions (both measured as the partition coefficient, *k*) achieved during two individual analyses is the principal to the compound identification in 2D-HPLC. Similarly, in 2D-TLC, the spots of two mixtures (plant extract and standards) are separated with the use of two chromatographic plates [3–5]. Subsequently, the sample components are identified by comparison of their retardation factor (*R*_*F*_) values with those of the separated standards.

The aforementioned technique has some drawbacks owing to the differences in conditions of the separation process onto two chromatographic plates, what may cause errors in identification of the components of the plant extract. It especially concerns compounds with similar polarity. Thus, zones of determined solutes may be contaminated with other compounds.

It is more rational to separate the plant extract and the mixture of standards parallel with the use of a single plate and identical conditions [6, 7]. The simultaneous separation of two samples (extract and the mixture of standards) applied onto the distinct ends of the chromatographic plate has been presented in [7]. Authors have separated both zones in two directions. The chromatographic plate containing solutes is developed in the first direction using a first mobile phase. Subsequently after eluent evaporation from the adsorbent layer, the chromatogram is developed in the perpendicular direction from two short sides of the plate (e.g., with the use of the horizontal chamber). After the second development, the separated extract and separated mixture of standards form mirror images. Both types of spots (separated sample compounds and standards) migrate identical distance from the line dividing the chromatogram onto two parts [7]. Such technique permits identifying the compounds of both samples by direct comparison.

The method described above sometimes fails to achieve complete separation of spots, especially those containing dominant and minor components. Moreover, there is poor separation of solutes of similar retention. This fact makes the qualitative and quantitative analyses of the mixture impossible.

Authors propose the modification of the above-mentioned method. The new technique leads to the preconcentration of the solute zones during two-dimensional plate developments and they are consecutively separated with the use of the mobile phase gradients and the progressive change of the development distance.

New method has been successfully verified by the separation of two plant extracts (*Juniperi Oleum* and* Thymi Oleum*).

## 2. Experimental

Chromatography was performed on 10 cm × 20 cm glass HPTLC plates coated with silica gel Si 60 F_254_ (Merck, Darmstadt, Germany). Essential oils (*Juniperi Oleum* and* Thymi Oleum*) were obtained from Profarm Sp. z O.O. (Lębork, Poland) and dissolved in toluene to furnish 0.2% v/v solutions.

The organic solvents (toluene, ethyl acetate, and methanol) and sulphuric acid were purchased from Avantor Performance Materials (Poland, Gliwice), while the vanillin was obtained from Merck (Darmstadt, Germany), whereas anise aldehyde was received from Sigma-Aldrich (St. Louis, MO, USA).

15 *μ*L of the toluene solutions of essential oils (*Juniperi Oleum* and* Thymi Oleum*) was applied as 1 cm zone on the 10 × 20 cm chromatographic plates by means of the Hamilton HPLC syringe.

### 2.1. Chromatographic Development

Chromatographic plates after spot applications were developed in a horizontal Teflon DS L or DS-II-10 × 20 chamber (Chromdes, Lublin, Poland) in the first direction in traditional way with mixture of toluene and ethyl acetate as the eluent. The development distances were varied from 6 to 10 cm. Then, the mobile phase has been evaporated. Subsequently, the plate was turned by 90°  and the separated zones were preconcentrated by development with a strong volatile solvent on the distance from 0.8 to 2 cm from the starting point. The chemical properties of investigated solutes influenced the type of eluent applied. Thus, the monoterpenes were developed with toluene or ethyl acetate, whereas more polar compounds, that is, polyphenols, required stronger eluent, that is, methanol or mixture of water and methanol. The preconcentration procedure was repeated until the zones were narrow and compact; after each repetition, the plate was dried. Then, the plate was developed in full distance in the perpendicular direction [8]. All experiments were conducted at room temperature (20°C).

### 2.2. Detection of Compounds

The spots of separated compounds were detected with anise aldehyde in sulphuric acid (0.5 mL of anise aldehyde, 10 mL glacial acetic acid, 85 mL methanol, and 5 mL 95% sulphuric acid) or with vanillin ethanol solution (1 g of vanillin, 100 mL 95% ethanol, and 10 mL 95% sulphuric (VI) acid).

The plate was heated to 100°C until the colour spots became visible.

The spots were detected with Desaga (Heidelberg, Germany) CD 60 densitometer (slit size 0.2 mm × 2.0 mm; *λ* = 560 nm).

## 3. Results and Discussion

The new method, presented in experimental part procedure, has been applied to separate compounds of two essential oils, that is,* Juniperus* and* Thymi*. According to the literature, both investigated mixtures contain solutes of various polarities (e.g., monoterpenes, sesquiterpenes, and monoterpene alcohols) [9–11]. Various chromatographic techniques, that is, GC [12] or TLC with special detection mode (BioArena [10], MS [9], and bioautography [11]), have been employed for determination of* Juniperus* and* Thymi* oil compounds. However, till today, there is no information on applying the multiple gradient development for this purpose.

**Figure 1:**
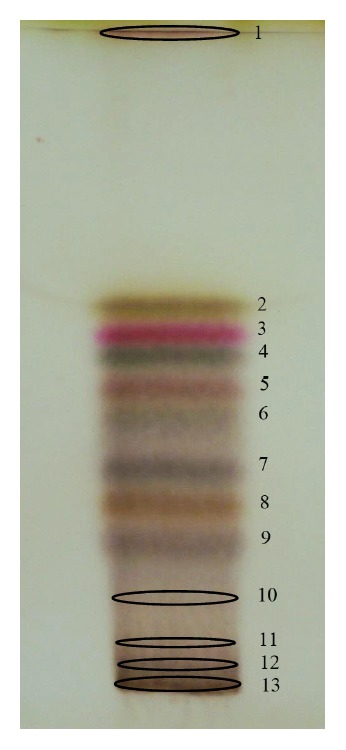
1D separation of* Juniperi Oleum* compounds. The mobile phase: toluene and ethyl acetate (94 : 6 v/v). The zones were derivatized with the anise aldehyde-sulphuric (VI) acid reagent.

**Figure 2:**
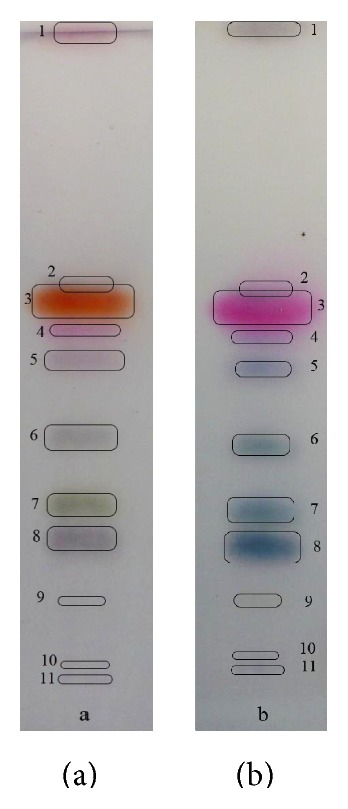
The 1D-TLC separation of* Thymi Oleum* compounds. The mobile phase: toluene and ethyl acetate (97.5 : 2.5 v/v). The two modes of compounds derivatization: (a) anise aldehyde-sulphuric (VI) acid reagent and (b) vanillin in ethanol reagent.

The plate containing the zones is developed in the first direction with isocratic elution (mixture of toluene and ethyl acetate) at the first stage of experiments.* Juniperi* oil contains essential amount of monoterpene alcohols [9] which requires more polar mobile phase (toluene and ethyl acetate; 94 : 6 v/v) in comparison to the* Thymi* oil (toluene and ethyl acetate; 97.5 : 2.5% v/v). The results of separation of* Juniperi Oleum* and* Thymi Oleum *are presented as photos in Figures 1 and 2, respectively. However, the purity of separated zones is unknown. Therefore, the development of the chromatogram in the perpendicular direction with gradient of the mobile phase is undertaken. The development programs for eluents realized with MGD-2D TLC have been determined experimentally and are presented in Tables 1 and 2 for* Juniperi Oleum* and for* Thymi Oleum*, correspondingly. Decreasing of the separation distances and simultaneously increasing of the mobile phase polarity during the multiple plate developments enhance the separation of the polar compound zones on one hand and keep nonpolar compound zones separated, on the other hand.

**Figure 3:**
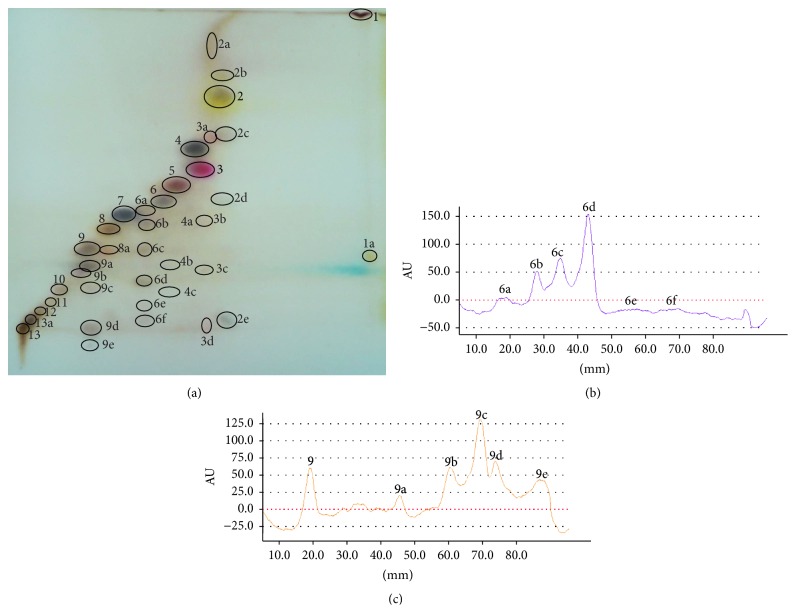
(a) MGD-2D TLC separation of* Juniperi Oleum* compounds. The program of eluent for second dimension development is presented in Table 1. The spots were derivatized with the anise aldehyde-sulphuric (VI) acid reagent. (b) Densitogram of the separation of compound 6 (*R*_*F*_ = 0.43 from isocratic elution) into additional bands by means of MGD-2D-TLC. Peaks with 6e and 6f were not visible with the applied wavelength (560 nm). (c) Densitogram presenting the separation of compound number 9 (*R*_*F*_ = 0.23, from isocratic elution) into additional bands by means of MGD-2D TLC. Peaks with low *R*_*F*_ values, not marked on the densitogram, were not visible with the naked eye. Their detection was performed by densitometry at the wavelength of 560 nm (compare Figures 1 and 3).

The separation of* Juniperi Oleum* with MGD-2D-TLC technique is presented in Figure 3(a). The application of new mode of development leads to achieving five additional zones of compound number 6 from isocratic system (Figure 1) denoted as 6a, 6b, 6c, 6d, 6e, and 6f (densitogram, Figure 3(b)). Also, compound number 9 from one-dimensional development (Figure 1) turns to be the mixture since five additional spots are detected (see densitogram in Figure 3(c)).

**Figure 4:**
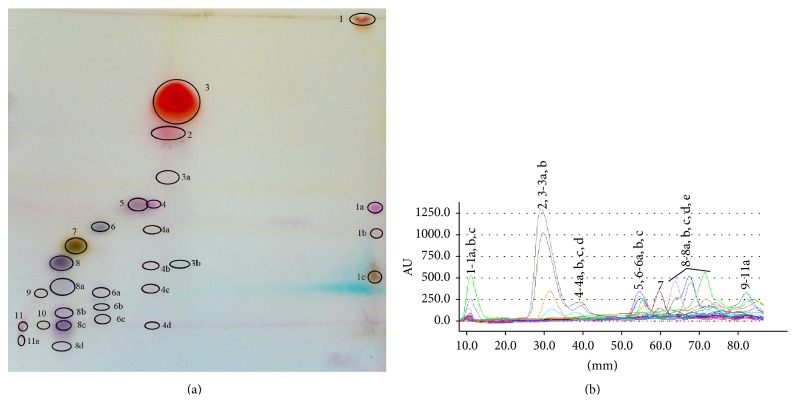
(a) MGD-2D TLC chromatogram presenting the separation of* Thymi Oleum* compounds. The eluent gradient program for second dimension development is presented in Table 2. Solutes were derivatized with the anise aldehyde-sulphuric (VI) acid reagent. (b) Overlaid densitograms of the* Thymi Oleum* separations into additional bands by means of MGD-2D TLC. The eluent gradient program for second dimension development from Table 2.

Correspondingly,* Thymi Oleum* is analysed with the new technique. The enhancement of the zone numbers is presented in Figure 4(a). Some of the identified solutes from one-dimensional development (Figure 2) such as compounds number 3 (thymol, *R*_*F*_ = 0.61), number 4 (1,8-cyneole, *R*_*F*_ = 0.55), number 6 (linalool, *R*_*F*_ = 0.37), and number 8 (borneol, *R*_*F*_ = 0.24) are separated into additional zones (number 3 into 3(a) and 3(b); numbers 4 and 8 into 4 extra zones; number 6 into 6 extra zones). The only pure zone turns to be carvacrol (compound number 2). Overlaid of* Thymi Oleum* separation densitograms obtained with MGD-2D TLC technique is presented in Figure 4(b).

The essential differences between single, isocratic development and MGD-2D-TLC of investigated oils are summarized in Tables 3 and 4. The application of MGD-2D TLC to* Juniperi Oleum *separation brings about the enhancement of number of separated spots from 13 (from 1D, isocratic development) into 34. What is more, the same effect was observed for the second separated oil. Only 11 zones were obtained by simple isocratic TLC separation of* Thymi Oleum* while in the new technique the number increased to 31.

The detailed description of the novel MGD-2D TLC technique has been published in Polish Patent [8].

## 4. Conclusions

The successful application of the above-mentioned technique for separation of* Juniperi Oleum* and* Thymi Oleum* as model complex mixtures makes it promising to further investigations. Continuation of this study will consist in spectrophotometric estimation of purity of separated zones.

The presented results indicate that a determination of the purity of separated zones in planar chromatography cannot be based on a simple isocratic technique. Application of two-dimensional multiple gradient development leads to the isolation of minor compound in the presence of dominant one. Additionally, changes of the development distances make this technique promising for separation of polar compounds. New method enables also more reliable estimation of the pharmacological properties of the components (e.g., lipophilicity). What is more, compact, concentrated zones of solutes may be further investigated with the use of various detectors, for example, mass spectrometry or diode array spectrophotometry.

## Figures and Tables

**Table 1:** The program used for four-step gradient elution for the development in second dimension for *Juniperi Oleum*.

Mobile phase	Step number 1	Step number 2	Step number 3	Step number 4
Toluene	99.0	97.0	96.0	92.0
Ethyl acetate	1.0	3.0	4.0	8.0
Development distance (cm)	10.0	9.5	8.0	6.0

The composition is given in % v/v.

**Table 2:** The program used for five-step gradient elution for the development in second dimension for *Thymi Oleum*.

Mobile phase	Step number 1	Step number 2	Step number 3	Step number 4	Step number 5
Toluene	100.0	99.0	98.0	97.0	97.0
Ethyl acetate	0.0	1.0	2.0	3.0	3.0
Development distance (cm)	10.0	9.0	9.0	8.0	8.0

The composition is given in % v/v.

**Table 3:** The comparison of efficiency of* JuniperiOleum *separation by means of two methods: isocratic elution and MGD-2D TLC. Compounds applied in order of decreasing *R*_*F*_ value and increasing polarity.

Isocratic elution		MGD-2D TLC
Number of band	*R*_*F*_	
1	0.99	Band number 1 → 1a
2	0.68	Band number 2 → 2a, 2b, 2c, 2d, 2e
3	0.59	Band number 3 → 3a, 3b, 3c, 3d
4	0.53	Band number 4
5	0.46	Band number 5
6	0.43	Band number 6 → 6a, 6b, 6c, 6d, 6e, 6f
7	0.34	Band number 7
8	0.24	Band number 8 → 8a
9	0.23	Band number 9 → 9a, 9b, 9c, 9d, 9e
10	0.14	Band number 10
11	0.06	Band number 11
12	0.04	Band number 12

**Table 4:** The comparison of efficiency of* ThymiOleum *separation by means of two methods: isocratic elution and MGD-2D TLC. The identification of particular compounds was performed according to *R*_*F*_ values given in the literature.

Isocratic elution			MGD-2D TLC
Number of band	*R*_*F*_	Compound	
1	0.99	Unknown	Band number 1 → 1a, 1b, 1c
2	0.63	Carvacrol	Band number 2
3	0.61	Thymol	Band number 3 → 3a, 3b
4	0.55	1,8-Cyneole	Band number 4 → 4a, 4b, 4c, 4d
5	0.49	*α*-Terpineol	Band number 5
6	0.37	Linalool	Band number 6 → 6a, 6b, 6c, 6d, 6e, 6f
7	0.26	p-Cymene	Band number 7
8	0.24	Borneol	Band number 8 → 8a, 8b, 8c, 8d
9	0.11	Unknown	Band number 9
10	0.04	Unknown	Band number 10
11	0.01	Unknown	Band number 11 → 11a
